# A generic solution for web-based management of pseudonymized data

**DOI:** 10.1186/s12911-015-0222-y

**Published:** 2015-11-30

**Authors:** Ronald Lautenschläger, Florian Kohlmayer, Fabian Prasser, Klaus A. Kuhn

**Affiliations:** Chair for Biomedical Informatics, Department of Medicine, Technical University of Munich (TUM), Grillparzerstraße 18, 81675 Munich, Germany

**Keywords:** Electronic data capture, Security, Privacy, Confidentiality, Pseudonymization, Web-based application, Seamless integration, Mashup, Cross-domain communication

## Abstract

**Background:**

Collaborative collection and sharing of data have become a core element of biomedical research. Typical applications are multi-site registries which collect sensitive person-related data prospectively, often together with biospecimens. To secure these sensitive data, national and international data protection laws and regulations demand the separation of identifying data from biomedical data and to introduce pseudonyms. Neither the formulation in laws and regulations nor existing pseudonymization concepts, however, are precise enough to directly provide an implementation guideline. We therefore describe core requirements as well as implementation options for registries and study databases with sensitive biomedical data.

**Methods:**

We first analyze existing concepts and compile a set of fundamental requirements for pseudonymized data management. Then we derive a system architecture that fulfills these requirements. Next, we provide a comprehensive overview and a comparison of different technical options for an implementation. Finally, we develop a generic software solution for managing pseudonymized data and show its feasibility by describing how we have used it to realize two research networks.

**Results:**

We have found that pseudonymization models are highly heterogeneous, already on a conceptual level. We have compiled a set of requirements from different pseudonymization schemes. We propose an architecture and present an overview of technical options. Based on a selection of technical elements, we suggest a generic solution. It supports the multi-site collection and management of biomedical data. Security measures are multi-tier pseudonymity and physical separation of data over independent backend servers. Integrated views are provided by a web-based user interface. Our approach has been successfully used to implement a national and an international rare disease network.

**Conclusions:**

We were able to identify a set of core requirements out of several pseudonymization models. Considering various implementation options, we realized a generic solution which was implemented and deployed in research networks. Still, further conceptual work on pseudonymity is needed. Specifically, it remains unclear how exactly data is to be separated into distributed subsets. Moreover, a thorough risk and threat analysis is needed.

**Electronic supplementary material:**

The online version of this article (doi:10.1186/s12911-015-0222-y) contains supplementary material, which is available to authorized users.

## Background

While collaborative research is developing rapidly, (e.g. [1–4]) a series of publications has shown relevant privacy threats [5], especially when genomic data are involved [6, 7]. On the other side, security of research data and biosamples is being addressed by regulations. The most important are the European Directive on Data Protection [8] (which is currently undergoing a reform process [9]), the European Recommendation on Research on Biological Materials of Human Origin [10] and the HIPAA Privacy Rule [11].

On the technical and organizational level, state-of-the-art security measures are needed to protect sensitive research data from unauthorized access. Important techniques include the use of secure network communication, strong authentication mechanisms, role-based access and different access tiers.

### Definitions and scope

**Pseudonymization** adds an important layer of protection for person-related data. It has been implemented in many projects (e.g. by the UK Biobank [12], the Icelandic biobank run by deCode Genetics [13] and the German National Cohort [14]) and it has become an important security measure required by laws and regulations. The term “separation” plays a central role in various definitions and regulations. The formulation in the Proposal for a General Data Protection Regulation of the Council of the European Union [9] is: “personal data may be processed for […] scientific research purposes only if […] data enabling the attribution of information to an identified or identifiable data subject **is kept separately** from the other information”, and in the German Federal Data Protection Act [15]: “characteristics enabling information concerning personal or material circumstances to be attributed to an identified or identifiable individual **shall be stored separately**”, and in the Italian Personal data protection code: “identification data **shall be stored separately** from all other data“[16]. There are, however, different definitions of pseudonymity and even synonyms for the term itself (including “coding” and “aliasing” [15, 17]). For the purpose of this work, we will use the term pseudonymity according to the description by Kalra et al. (who in turn cite a definition by Lowrance [17]) [18]: “Pseudonymization (reversible anonymization, or key coding) involves **separating** personally identifying data from substantive data but maintaining a link between them through an arbitrary code (the key).”

The ISO Technical Specification 25237 on “Health informatics - Pseudonymization” also addresses separation: “identifying and payload data shall be separated” [19]. While **separation** is a common element in the cited sources, there is no explicit specification of **what exactly** has to be separated. It is clear that separation will require at least two data pools. Kalra uses the term “identifying” data to characterize the **first** one, while the above regulations describe this first part as “data enabling the attribution […] to an identified or identifiable data subject”. In slight difference to Kalra, ISO 25237 uses the terms “**identifying**”, “quasi-identifying” or “indirectly identifying” [19]. We will refer to identifying data as **master data**. For the content of the **second** pool, Kalra uses “substantive data”, the regulations call it “other data”, and ISO uses the term “**payload**”. It is particularly unclear which attributes should (or can) remain in this second pool. Both ISO 25237 and Pommerening et al. [20] have addressed but not completely clarified this. Pommerening et al. [20] have introduced additional types of data: 1) identifying data, 2) medical- or clinical phenotype data, 3) data associated with the management of biospecimens, and 4) data resulting from the analysis of biospecimens. We will not further address the specifications of different types of data [19, 20], and consider data pools to be pre-defined. We recommend, however, clarification by further work. Our focus will be on separation and on the management of pseudonyms. We will address pseudonymous identifiers for biosamples, but we will not go into any detail of biosample management itself.

**Fig. 1:**
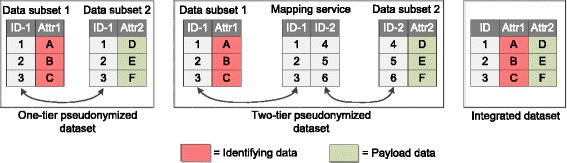
Examples for pseudonymizing datasets with data-layer separation. The example dataset consists of two attributes (Attr1 and Attr2) that are separated from each other. The first attribute can be considered identifying, whereas the second attribute contains payload data. The dataset contains three data entries

Some further clarifications are necessary. While anonymous data are not considered personal data in a regulatory sense, pseudonymous data remain personal data [9]. There is a distinction between irreversible and **reversible pseudonymity**: within this article, we will focus on the latter case. As separation of data is a core characteristic of pseudonymization, we illustrate it by Fig. 1, showing two different options.

Option 1, which we call “one-tier pseudonymized” has been used in trials for decades, the “two-tier pseudonymized” approach is more recent and it is recommended by ISO 25237 [19] and by Pommerening et al. [20]. Using the terminology from ISO 25237, we will consider A, B, C “identifying” and D, E, F as “payload”. Two-tier pseudonymity means that the datasets are interlinked via a set of cascading identifiers. When two-tier pseudonymization is used, each component maintains its own *namespace* for identifiers. The identifiers from different namespaces are linked by a dedicated mapping service. The figure also shows the “integrated dataset” that can be constructed by de-pseudonymizing the dataset. We will refer to this simplified data-centric view of Fig. 1 throughout this paper.

It is important to distinguish between concepts and their implementation. A **pseudonymization concept** describes a more or less abstract separation of data into different pools, potentially combined with specifications of valid data flows and implementation constraints. Already on the concept level, there are different approaches to pseudonymization. In this article, we will focus on two models: the ISO Technical Specification 25237 [19] on “Health informatics - Pseudonymization”, which describes concepts fundamental to pseudonymity in biomedical research environments, and the German model by Pommerening et al. [20], which is closely related to ISO 25237. Overviews of the concept by Pommerening et al. [20] can be found in [21–23]. The solutions described by Brinkmann et al. [24], Spitzer et al. [25], and Lablans et al. [26] are also based on the German model, and therefore these references contain short descriptions of the model.

### Objectives

Motivated by the need to design and implement IT solutions for several research projects [27–29], we had (1) to collect and systematize core requirements for secure solutions, (2) to compare implementation options, and (3) to implement a generic solution. The focus of our work is on multi-site research registries with sensitive biomedical data; the management of biosamples had to be addressed by the solution, but will only be shortly touched in this article.

## Methods

In order to get a conceptual basis, we have started our work with an analysis of two comprehensive concepts ([19, 20]). While ISO [19] is international by its definition, [20] is being considered a set of quasi-standard requirements in Germany. From these two sources, we compiled a set of fundamental requirements for pseudonymized data management. Functional requirements were taken from related work on electronic data capturing systems and complemented with results from our own analyses which we cannot describe in detail here. The next step was to design a system architecture fulfilling these requirements. For this purpose, we analyzed several architectural options. Then, we created an overview of technical options for an implementation and performed a comparison. The final step has been the implementation of a generic solution. Its feasibility has been demonstrated in research networks of which we will shortly describe two ([27, 29]). Institutional Review Boards (IRBs) and data protection officers of the participating sites have approved the concept. For a comprehensive list we refer to Additional file 1.

## Results

The methodical approach described above has led to results which we will present in the same order: (1) core requirements, (2) an analysis of architectural options, (3) a high-level system architecture, (4) an analysis of technical options, (5) a technical design and (6) several implementations.

### Requirements analysis

When implementing an electronic data collection system, the permission model for access to data has to be designed carefully, e.g. following the need-to-know principle as well as the principle of least privilege. Audit trails are essential in any case. Context-dependent rights and roles of users are an important factor: who (in which role) has the right and the need to know which data in which context. In general, a health care professional treating a patient may need more permissions than a researcher. We will not follow up on these aspects, because they are not directly related to the problem of pseudonymization.

Instead, we will focus on *functional requirements* between the system and its users that are affected by introducing pseudonymity and *non-functional requirements* that are implied by pseudonymization concepts. We note that this classification is in-line with the according concepts in software engineering: non-functional requirements may be defined as “not directly concerned with the specific services delivered by the system to its users” [30].

### Functional requirements

Our solution focusses on a clearly defined use case: collaborative prospective electronic collection of longitudinal person-related data. The key stakeholders are users and patients. Users are health care professionals, study nurses, registry monitors and researches. Functional requirements have been compiled from related work by Demiroglu et al. [31], Bialke et al. [32], Meyer et al. [33] and Spitzer et al. [25] and from a comprehensive overview by Ohmann et al. [34]. We complemented them by results from our own software engineering process, which we cannot describe in full detail here (see Kalman et al. [27] and Kohlmayer et al. [35] for a short overview). While links to biosamples play an important role in many systems (e.g. [27, 31, 35–37]), we will not cover this aspect in detail.

For our overview of functional requirements, we introduce a systematic order and focus on the subset of specific relevance to our topic. Where appropriate, we motivate functional system requirements with usage scenarios. The following set has resulted from our approach:

- **R-C1 - Data Collection:** The system shall support the collection of research data and metadata about associated entities in electronic forms or documents (eCRFs) [33–35].
- **R-C2 - Data Structuring:** Typically, different types of data are collected in different eCRFs that belong to the same context (e.g. patient or visit). The system shall provide means to maintain links between associated entities and documents. [38]
- **R-C3 –Integrated View:** The system shall support integrated views on different forms or documents between which an association exists [27, 32, 33].
- **R-C4 - Data Management:** The system shall provide methods for validation of data completeness and integrity [32, 34, 35].

We note that requirements *R-C1, R-C2, R-C3* and *RC-4* need a *legal basis* and must be covered by *informed consent.* We further note that *R-C3* may include an integrated view of master data and other types of data. This view should adhere to the need to know principle and must be compliant with legal and regulatory requirements. A usage scenario for R-C3 is the process of *re-contacting a patient or proband* in cases specified by patient information and informed consent. *R-C3* is also of relevance for *follow-up data collection* where (additional) information about a patient or proband needs to be entered during multiple visits. For this purpose, documents also need to be integrated with master data [27, 33, 35]. Finally, some processes of *data management* may also require an integrated view on several documents, for example, for cross validation [19].

### Non-functional requirements

Patients have an inherent interest in security and confidentiality of the data they have consented to share. At the same time, data management solutions for collaborative biomedical research has to be compliant with national and international laws. As outlined above, two pseudonymization concepts [19, 20] have been our basis for formulating non-functional requirements. We will present a set of non-functional requirements, which define a system that is able to fulfill the functional requirements while ensuring compliance with pseudonymization concepts. Were appropriate, we will motivate non-functional requirements with references to functional requirements and requirements implied by these concepts. To describe the central aspect of “separation”, we start by focusing on the data layer. Typically, information systems are described by further layers, comprising an application and a presentation layer [39], which support (and to some degree model) real-world processes [40]. We will structure non-functional requirements along these layers.

#### Requirements on the data layer

On the data layer, the concepts [19, 20] define pseudonymization of a dataset as a separation into subsets containing different types of data. The records within these subsets are stored in different locations and they are interlinked with identifiers. Data collection and management can be modeled as a set of *CRUD* operations on documents: (1) *Create*: creates a new document, (2) *Read*: provides a view of the data contained in one document or a list of other documents related to one document. (3) *Update*: provides a view of the data contained in a document while allowing updating its content. (4) *Delete*: deletes a document.

- **R-D1 - Distributed CRUD:** The system shall implement data collection on top of a set of distributed databases.
- **R-D2 - Physical separation**: The system shall support the hosting of different backends on different physical machines with different host names.
- **R-D3 - Two-tier pseudonymization**: The system shall provide support for two-tier pseudonymization, implemented with an additional mapping service.

As a result of *R-D1, operations on documents must be performed across different data pools*. Requirement *R-D2* is motivated by the fact that [19, 20] require the installation of *separate governance, duties and responsibilities* for the individual data pools. Requirement *R-D3* is motivated by our aim to provide a generic solution for both pseudonymization concepts [19, 20] which *require two-tier pseudonymity in several cases* (e.g. when biosamples are involved and/or in multi-site research networks).

#### Requirements on the application layer

As the application layer supports workflows that are provided to users, requirements on this layer are strongly influenced by the above functional requirements:

- **R-A1 – De-Pseudonymization:** The system shall support the de-pseudonymization of data.

On the documentation level, and thus on the system level, re-identification requires *reversing the separation* between identifying data and payload data [20, 31, 35]. De-pseudonymization, which equals a re-identification of data subjects, is a core element related to (reversible) pseudonymity. On the real-world level, re-identification means revealing the hidden identity of a subject. The non-functional requirement *R-A1 is implied by functional requirement R-C3*. The latter is motivated by several usage scenarios, for which a legal basis exists. We have summarized them above and they are described in detail in ISO 25237 [19].

Pommerening et al. have added the following requirements on application-layer:

- **R-A2 - Client-side re-combination**: The reconstruction of the logical global dataset shall only be performed at the client-side to reduce the number of attack vectors [20].
- **R-A3 - Confidentiality of internal identifiers**: Clients shall be unable to learn the pseudonymous identifiers used in the distributed databases [20].

#### Requirements on the presentation layer

- **R-P1 - Usability:** The system should *adhere to well-established usability guidelines* [34, 41].

This requirement *(R-P1)* is motivated by the fact that there are reports on systems in which the linkage of different data subsets must be performed manually, i.e., by copying and pasting an identifier displayed by the interface of one application into the interface of another application [26]. This process is time-consuming and error-prone [42]. Furthermore, *implementing a consistent user interface* for several distributed systems while ensuring a continuous workflow means that there should be no need for users to separately authenticate on the multiple systems involved.

#### Requirements on all layers

Data separation will inevitably lead to complex architectures which may negatively affect maintainability. We have therefore added the following requirement:

- **R-M1 - Maintainability:** The system should allow for centralized installation and maintenance [33, 35].

This requirement is quite typical for multi-site scenarios: the *integration of a system module into the security architectures of (distant) clinical or research sites* implies challenges such as managing institutional firewalls and software installation policies. Frequently, the system needs to support *a large set of users that are distributed geographically*.

### Analysis of architectural options

**Fig. 2:**
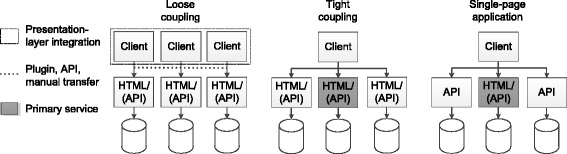
Design space for distributed data management. A loosely coupled architecture only provides little integration. A tightly coupled architecture integrates the interfaces from several backends. In single-page applications, the user interface is delivered by exactly one backend

Next, we have analyzed options to build an integrated interface to distributed databases. The architectural design space is shown in Fig. 2. In the remainder of this section we focus on applications built with web technologies. We note, however, that the presented system architectures are applicable to other development techniques as well.

### Loose coupling

The concept of *loose coupling* illustrates a thin layer implementing presentation-layer integration. In this case, users need to sequentially access different clients for separate systems, which might be displayed next to each other or be embedded into each other. Moreover, methods for context management, such as HL7 CCOW [43], may be used. Loose coupling does only support limited exchange of data between interfaces (*interface-to-interface communication*). Operations like creating, updating and deleting documents have to be performed manually, potentially repeatedly on the interfaces of the multiple systems over which the data of an entity is distributed. To maintain consistency, identifiers must often be transferred manually from one system to another. Obviously, this represents an error-prone and inefficient workflow, which may lead to data quality issues. Furthermore, using the system is complicated, as different modules may utilize different user interface designs and different interaction patterns, which may negatively affect user acceptance. Most papers describing implementations of pseudonymization concepts are based on the principle of loose coupling [26, 31, 32, 36, 37, 44, 45].

### Tight coupling

A design with *tight coupling*, which is also shown in Fig. 2, allows for integrated access to several endpoints. Here, each endpoint provides its own graphical user interface but a dedicated component (called the *primary service*) delivers the main application and provides presentation-layer integration of user interfaces. Moreover, endpoints may provide additional programming interfaces for access to data. These access points may be used by the central component to enable interface-to-interface communication, resulting in a seamless user experience. In contrast to loosely coupled designs, the central component needs to process and display data from different domains. Moreover, business logic is more complex because access and interaction between the separated services must be orchestrated.

### Single-page application

In contrast to tight coupling, a *Single-page Application* (SPA), which is also sketched in Fig. 2, does not implement any form of presentation-layer integration but only uses a single graphical interface provided by one endpoint together with further interfaces for accessing data from the other endpoints. As a consequence, the backends only need to implement very little business logic. Access to backends is, e.g., provided via a *Web Application Programming Interface* (Web API). Typical examples include *Representational State Transfer* (REST) interfaces or *Web Services* (WS) using *JavaScript Object Notation* (JSON) or *Extensible Markup Language* (XML) as a syntax for message exchange. Such applications can be implemented with client-side *Model-View-Whatever* (MVW) frameworks, such as, *AngularJS* [46] or *Backbone.js* [47].

### Architectural design

Based on the requirements described above and the architectural options identified, we designed a high-level system architecture. To reduce installation efforts and ensure compatibility with enterprise security architectures, we decided to implement a *web-based system* that adheres to established web standards and is thus accessible from a broad spectrum of web browsers. Moreover, distributing updated versions of our software becomes easy (*R-M1*).

On an architectural level, we decided against a loosely coupled design due to the problems described above. For a Single-Page Application the technologies supported by legacy web browsers are insufficient. Frameworks for SPAs are partially immature and not in widespread use. Hence, we decided for a *tightly coupled architecture* that guarantees seamless integration (*R-A1*) and good usability (*R-P1*) based on reliable and widely supported technologies.

The requirement that it must only be possible to re-construct the dataset at the client side (*R-A2*) is fulfilled by employing *client-side mashup-techniques*. In short, a client-side mashup displays data from different servers in an integrated manner within a user’s local browser. To support multi-tier pseudonymity (*R-D3*), we maintain a *mapping service*, which translates pseudonymous identifiers from the namespace of one system into the namespace of another. It is ensured that the distributed datasets can only be joined at the client systems by exclusively delivering data to clients, meaning that no data is (directly) exchanged between backend services. In this process it is further ensured that clients cannot learn pseudonymous identifiers (*R-A3*) by *substituting identifiers* within the distributed datasets with *temporary identifiers* before delivering any data. To allow for a re-combination of separated data subsets these temporary identifiers must be synchronized between the backend services. To this end, a *secure server-to-server communication channel* is needed that is not accessible by clients. To ensure consistency while supporting common types of database operations (*R-D1*), data is managed in a set of distributed *relational database management systems* (RDBMSs).

Two problem-specific challenges arise from the design decisions described above. Firstly, to ensure continuous workflow, a *Single-Sign-On (SSO)* mechanism has to be implemented (*R-P1*). Secondly, in all modern browsers, the implementation of client-side mashups of data retrieved from different domains is complicated by the *Same-Origin-Policy (SOP)*. The basic principle of the SOP is that “only the site that stores information in the browser may later read or modify that information” [48]. This security feature prohibits cross-domain communication, which, on the other hand, is required to re-integrate distributed data subsets that must be hosted on different physical machines in our setup (*R-D2*).

### Analysis of technical options

In order to proceed from a high-level architecture towards an implementation, a variety of implementation options for different aspects of the architecture exists and has to be discussed. In this section, we will present and compare several options for implementing the most important modules of the system: (1) client-side web mashups, (2) single-sign-on mechanisms, and, (3) methods for providing a secure server-to-server communication channel.

### Web mashups

In [49] a mashup is defined as “a website […] that seamlessly combines content from more than one source into an integrated experience”. In the context of our work, implementing a Web Mashup is challenging, because data is stored in different physical locations and thus accessed via different interfaces provided by servers from different fully qualified domain names. Integrating such distributed interfaces and data conflicts with the *Same-Origin Policy* (SOP), a security feature which prohibits cross-domain communication. It was designed to protect a user’s privacy by preventing sites from tracking a user’s behavior, e.g., by reading stored cookies or data from the cache. The SOP also prevents a user’s actions from being corrupted by other websites and it prevents websites from performing transactions on behalf of the user [49]. The SOP is implemented by only allowing scripts to modify a web page of the same origin only (i.e., loaded by the browser from the same domain). The work by De Ryck et al. presents an overview of state-of-the-art mashup techniques [50]. Well-known techniques that can be used to realize mashups include *HTML Frames*, *PostMessage*, *XMLHttpRequest* (XHR) and *JSON with Padding* (JSONP) [50]. However, not all of these techniques provide means to circumvent the restrictions implied by the SOP.

#### HTML frames

An HTML-frameset is a group of HTML-frames. The content of a frame is dynamically loaded and independent of the other frames in a frameset. IFrames (inline frames) were introduced in HTML 4.0. In contrast to standard HMTL-frames, IFrames allow for embedding HTML-documents in the body of other HTML documents. HTML-Framesets and IFrames can be used to display contents from different domains in a browser but without supporting any kind of interaction. Enforcing the SOP, the contents of each origin will be loaded separately and isolated from the contents of other frames.

#### PostMessage

The HTML postMessage mechanism enables cross-domain communication by enabling scripts to send messages to HTML Frames or windows of arbitrary origin. This feature, available since HTML 5, relies on the recipient to verify that the message is from a valid or authorized sender [51]. It is not supported by legacy browsers.

#### XMLHttpRequest (XHR)

XHR is a widely supported API in web browsers that allows sending HTTP requests to a server which returns XML-, TEXT/HTML- or JSON-formatted data. It is accessed with web scripting languages, most commonly JavaScript. XHR does not support cross-domain communication unless the client supports Cross-Origin Resource Sharing (CORS), which is a W3C recommendation since early 2014 and is therefore not supported by legacy browsers.

#### JSON with Padding (JSONP)

JSONP is a communication technique that exploits the fact that the SOP is not applied to the src-attribute of an HTML script tag. Here, a script tag is created in which the endpoint from which data is to be received is defined as the src-attribute. To make the data available to the local scripting context, the endpoint embeds the requested data into a call to a local JavaScript function. Which function is to be called with the requested data is encoded into the Query String of the endpoint’s URL. The name JSONP stems from the fact that data is typically encoded in JSON format and can thus be directly transformed into JavaScript objects.

#### Server-side mashups

A server-side mashup can be implemented by employing a proxy that integrates data from different sites into a common context and delivers it to the clients. A proxy can also be used to mask different origins of data and thus circumvent the SOP [49]. In our context, server-side mashups cannot be used. A proxy must be able to see all the information that it has to integrate. On the other hand, sensitive personal data managed by research systems must only be transported via encrypted channels (typically using Transport Layer Security (TLS/SSL)). Additionally, this encrypted channel must be established between the client and the data stores, because of the requirement to restrict the context of data linkage to the local machines of users.

### Single-sign-on

A web mashup must be combined with a Single-Sign-On mechanism that ensures a continuous workflow by making it unnecessary for users to separately authenticate on the multiple systems involved. In addition, a complex system for collaborative research also requires means for authorization. The same design decisions that must be made for authentication must also be made for authorization: (1) should the according mechanism be implemented by a dedicated component within the distributed system (e.g. using Shibboleth for authentication), or (2) should each component handle the according aspect by itself. In this section we provide an overview of these design dimensions and present several techniques that can be used to implement the various aspects involved.

#### Non-delegated authentication

In this setup, each component handles authentication by itself. The most straight-forward implementation simply includes the user’s credentials (user name and password) in all requests to an endpoint. When, as in our case, servers are stateful, a server-side session must additionally be associated with the client. Sessions are usually identified by a randomly generated token and these IDs can (and will) thus be different for different sessions at different servers. As a result, the client must either actively manage a set of session IDs, one for each server, within its business logic or use a passive approach, such as cookies.

#### Cookies

While not directly related to authentication and authorization, cookies are a widespread technique to make user sessions persistent across several requests to an endpoint. Here, the unique session ID is stored in a local file (called cookie), which is transparently transferred to the host on every request. Because the Same-Origin-Policy also applies to cookies (a single cookie cannot be sent to multiple endpoints hosted on different domains), this mechanism cannot be used to implement cross-domain Single-Sign-On. However, cookies can complement SSO solutions, because they can be used to persist individual sessions at different endpoints.

#### Server-to-server communication

Single-Sign-On can also be implemented with server-to-server communication. Here, opening a session at one endpoint transparently creates sessions on the other endpoints as well by implementing a communication mechanism between servers. As a result, it (a) can be ensured that a single user session is identified by the same token on different endpoints, and, (b) there is no need to send the user’s credentials to the endpoints with every request. This technique can be implemented, e.g., with a multicast protocol such as JGroups [52], sockets or a shared file system. Such an approach is difficult to integrate into Enterprise Security Architectures.

#### Access tokens

Typically, SSO solutions are implemented with cryptographic access tokens. Basically, a token is an object that encapsulates the identity and potentially roles of a user as well as a session ID. Tokens generated by one system can be used to perform operations on another system. A token can (and must) be validated by the target system. From a conceptual perspective, using access tokens is not different from the server-to-server communication approach. The only difference is that with the former approach server-to-server communication is *indirect*, i.e., performed via the client. This makes this approach feasible for implementing SSO between several isolated services on the World Wide Web. Consequently, the approach is, e.g., implemented by Kerberos [53] and Shibboleth [54]. Access tokens provide a secure communication channel between servers, meaning that the client cannot read or modify the content of a token. This is especially useful in our scenario, because it can be used to fulfil an additional non-functional requirement. When implementing access tokens, the main challenges are (1) transferring tokens from the clients to the server, and, (2) key management.

#### Rights and roles

The handling of authorization of a user’s actions is typically coupled with authentication. As a consequence, the design space is closely related to the design space for Single-Sign-On solutions. Role-based access control (RBAC) is an authorization mechanism in which rights are granted to users depending on their associated roles. A role encapsulates a set of permissions. Analogously to SSO, RBAC can be realized with a) a centralized component that authorizes users as well as b) a decentralized solution where every system implements a RBAC component and manages authorization by itself. Important standards for authorization in distributed environments include SAML and XACML [55].

### Secure server-to-server communication

For synchronizing temporary pseudonyms between backend services, secure communication channels are needed. In this context, secure means that the contents of messages are hidden from the clients. This can be implemented with two different mechanisms. Firstly, backend servers can manage exclusive communication channels between them and use these to synchronize information about temporary pseudonyms. Secondly, a secure channel between servers can be built that is routed through the client by using cryptographic tokens.

#### Direct communication

**Fig. 3:**
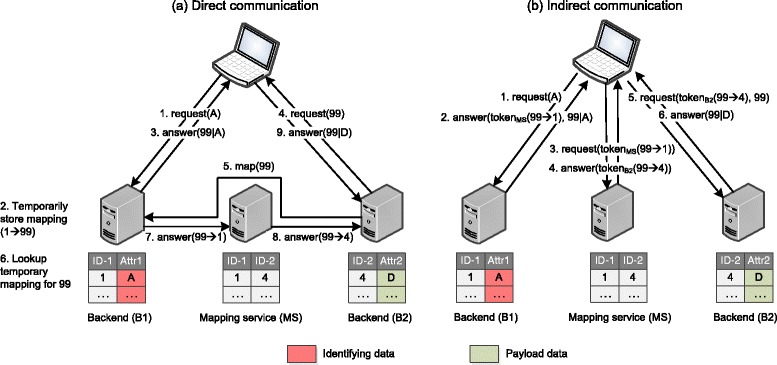
Two basic methods for joining distributed data with temporary identifiers and a mapping service. When using direct communication, backend servers synchronize temporary identifiers directly with each other. When using indirect communication, messages are exchanged via the client only. This communication pattern is less complex and servers remain stateless

Figure 3a shows how the reconstruction of a pseudonymized dataset using temporary identifiers can be performed with direct server-to-server communication. In step 1, the client requests a data item (A) from backend B1. The backend creates a temporary pseudonym for the data entry and persists its association to the actual identifier from its namespace (step 2). The data entry with substituted identifier is then delivered to the client (step 3). Next, the client requests the data item associated with the temporary identifier (step 4) from backend B2. In step 5, the backend requests a mapping of the temporary identifier from backend B1. B1 resolves this request by looking into its set of persisted temporary mappings (step 6). The answer to B2 must be routed through the mapping service (steps 7 and 8). Finally, in step 9, B2 delivers the data entry to the client. Problems with this approach include that (a) it is unclear when exactly the persisted substitution of an identifier may be deleted without implementing complex protocols for transactional guarantees, and, (b) at least seven messages must be exchanged to recombine data distributed amongst two databases via mapping service.

#### Indirect communication

Figure 3b shows the reconstruction of a pseudonymized dataset with indirect server-to-server communication. Analogously to the previous example, the client requests a data item from backend B1 (step 1). In step 2, the backend creates an association with a temporary identifier, replaces the actual identifier for the data item and sends it back to the client. In contrast to the previous scenario, where the mapping from the actual identifier to the temporary pseudonym is persisted, B1 also sends an encrypted token containing the association. The client forwards the token to the mapping service (step 3) where it is decrypted and the ID from backend B1 is translated in the associated ID at backend B2. Next, the mapping service generates a second token for B2, containing the mapping from the temporary pseudonym to the original identifier. This token is sent to the client (step 4) where it is forwarded to backend B2 (step 5). Finally, in step 6, B2 decrypts the token, performs a lookup for the data item and sends the result back to the client, along with the temporary pseudonym. At the client side, the data from both backends can be joined using the temporary identifier. We note that in this simple example, it would be sufficient to keep track of the relationships between requests and responses to perform a mapping of the contained data. In more complex real-world scenarios, however, tokens may contain multiple data entities. As a consequence, tokens must contain identifiers that allow combining individual data items from different backends. The use of temporary identifiers for this purpose is motivated by R-A3, which requires internal identifiers to be kept confidential. Compared to direct server-to-server communication, the number of exchanged messages is reduced. Fewer communication channels must be managed, because the same communication channels are used for client-to-server and server-to-server communication. Moreover, as already noted above, indirect server-to-server communication can also be implemented relatively easily, if access tokens are already used for implementing Single-Sign-On. In the remainder of this section, we will elaborate on ways to implement cryptographic (access) tokens.

#### Transferring tokens

While tokens can easily be sent from servers to clients in our context, sending tokens from clients to servers is more challenging. Tokens can be embedded into three different segments of an HTTP request: (1) HTTP request line (URL), (2) HTTP header fields, (3) HTTP message body. These techniques have different properties in terms of compatibility to legacy browsers, implementation complexity and compatibility with other techniques required to implement pseudonymized data management, especially JSONP. Here, all parameters must be encoded into a request URL, because, by specification, requests embedded into src-attributes are executed as HTTP GET-Requests by browsers. The only approach that ensures backwards compatibility and that is compatible with JSONP is embedding tokens into the HTTP request line via *URL Rewriting*. An additional challenge when implementing this method is to overcome a length restriction that is enforced to URLs by most browsers (e.g., 2083 characters per URL in Internet Explorer). This can be solved by implementing packet fragmentation mechanisms, i.e., splitting a request into multiple sub-requests at the client-side, which are recombined into one request at the server-side [56]. Tokens can also be embedded into the *header* of HTTP GET- and POST-Requests but this method can only be realized with JavaScript calls and is thus not compatible with JSONP. Finally, tokens can be embedded into the *HTTP Body* of POST-Requests. Again, this is not compatible with JSONP, because JSONP requires GET-Requests to be performed.

#### Key management

To maintain confidentiality for the contents of a token, symmetric or asymmetric (or hybrid) cryptography can be employed. Depending on the topology of the infrastructure (hierarchical or peer-to-peer), encryption also affects key management. In a hierarchical infrastructure, a single component can be employed to manage all keys needed for the encryption of tokens, whereas in a peer-to-peer infrastructure each component needs to manage key pairs for every other component. In web-based applications, tokens can be implemented with JSON Web Tokens (JWT) utilizing related technologies such as JSON Web Encryption (JWE), JSON Web Signatures (JWS) and JSON Web Keys (JWK) [57].

### Technical design

Based on requirements, we selected a set of technical options for an implementation. In this section, we will describe the resulting generic solution.

The maintainability requirement (*R-M1*) was weighted high in our implementation. Our aim was to develop a solution that is robust while only relying on technologies supported by common web browsers (including wide-spread legacy browsers). We therefore decided to build a client-side Web Mashup with HTML Frames for parts of the application that do not require any interface-to-interface communication and with utilization of JSONP for all other cases. In our implementation, data is modeled as a tree-like structure where the root represents a subject’s master data and further nodes represent documents containing payload data. To support data collection, as defined by *R-C1*, we realized interfaces for CRUD operations on this tree with two functional views: “*create, list & delete”* and “*view & update”*. The former provides a list of documents and allows creating new or deleting existing documents. The latter shows the content of a document and allows updating it. Several instances of these two types of views may be displayed next to each other, thus providing an integrated interface as required by *R-A1* to fulfill our functional requirements *R-C2*, *R-C3* and *R-C4*. JSONP is a good solution for interfaces in which data of many entities has to be displayed, i.e. the “*create, list & delete”* view. In the other cases, i.e. the *“view & update”* interface, we leverage HTML Frames because of their ease of implementation and therefore increased productivity when developing the software.

For Single-Sign-On (see *R-P1*) our solution implements non-delegated authentication where each component handles authentication and authorization autonomously. This design decision is driven by the fact that many pseudonymization schemes require at least one trusted third party (TTP), which is organizationally and physically separated from the rest of the system. As a result, decentralized authentication and authorization is performed for each request and each component provides its own administrative interface and RBAC model. Keys are distributed in a peer-to-peer topology. Non-delegated authentication is implemented with cryptographic access tokens that also provide a secure communication channel between servers that is routed via the client. Tokens are created by backend servers. At the client side, they are always appended to the URL. When using JSONP this is implemented with JavaScript, otherwise a server-side URL rewriting mechanism is used. Session-IDs are persisted with cookies. As defined in requirement *R-D3*, our solution supports two-tier pseudonymization. This makes joining distributed data more complex, because pseudonyms need to be translated from one namespace into another namespace. Moreover, requirement *R-A2* specifies that this linkage must only be performed at the clients. We use the same token infrastructure for SSO and for implementing an indirect communication channel between backend servers (cf. Section “Secure Server-To-Server Communication” and Fig. 3b).

There are multiple frameworks for implementing token infrastructures, but we decided to develop our own solution that is tailored to our requirements for the following reasons. JSON Web Tokens are still in a draft-phase and currently immature. XACML and SAML come with a significant overhead regarding the size of the exchanged messages because they use an XML-Syntax. This is problematic when transmitting data via URLs. Furthermore, XACML and SAML are complex, resulting in a rather high implementation effort. In our system, tokens are encrypted with a hybrid method combining AES and RSA. The payload is encrypted symmetrically and integrity protected and the key for decryption *K*_*1*_ is encrypted asymmetrically with the public key *K*_*2*_ of the receiver. Tokens contain the key *K*_*1*_*, username, password, counter* and *payload data P* (e.g. encoded in JSON syntax). In the following *E*_*x*_*(y)* denotes the encryption and integrity protection of *y* using the key *x*. The token is built of two components. The first component contains the key *K*_*1*_, which is encrypted and integrity protected with the public key of the Server (*K*_*2*_), i.e. *E*_*K2*_*(K*_*1*_). The second component contains the username, password, counter and the payload encrypted and integrity protected with the key *K*_*1*_ from the first component, i.e. *E*_*K1*_*(username, password, counter, P)*. Replay protection is implemented with a counter that is continuously incremented and prevents repeated acceptance of tokens by any receiver.

**Fig. 4:**
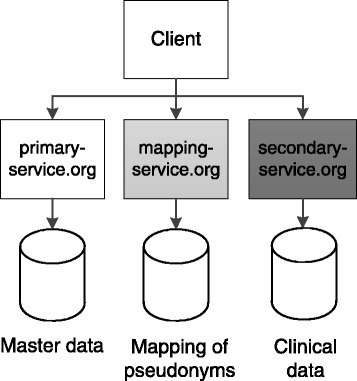
System architecture for a tightly coupled system with pseudonymization. The example shows the separation and two-tier pseudonymization of two data subsets. The data subsets as well as the mappings between pseudonymous identifiers are managed by backends with different host names

The design of our solution supports two or more physically distributed data stores (*R-D2*) and one or more mapping services (*R-D3*). All endpoints have to provide API access and all services but the mapping service must be able to provide HTML-formatted data to clients as well. However, the mapping service must provide HTML-Frames that embed HTML-formatted data from other services, as will be explained below. A basic design fulfilling all requirements of the model by Pommerening et al. [20] must implement separation of master data and clinical data [15]. A minimal solution is shown in Fig. 4. The central component is implemented by the backend managing master data (primary service), because it stores the root nodes of the tree and is thus the starting point for user interactions.

**Fig. 5:**
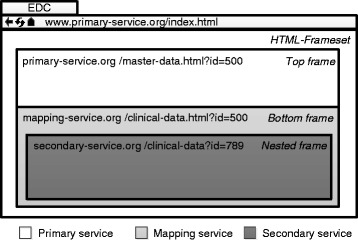
Presentation-layer integration of pseudonymized data with nested HTML Frames. In this mockup, interfaces provided by different backends are integrated by rendering them into nested HTML Frames. This enables the integration of a mapping service and thus the transparent resolution of pseudo-nymized relationships

Our final solution combines the above techniques into a Web-Mashup that integrates pseudonymized data (*R-A2*). The first variant, which uses HTML-Framesets, is sketched in Fig. 5. Here, a static frame at the top displays selected data of a single entity from the primary service. The content of the second frame, which is located at the bottom, is provided by the mapping service and contains an additional nested frame, which shows the corresponding clinical data. Please note that in Fig. 5 pseudonyms are represented as clear text instead of being encoded into tokens for the sake of readability. In our implementation pseudonyms are encoded into encrypted tokens and therefore never visible to the client (*R-A3*).

A typical workflow in which the above method is utilized is the creation of a new eCRF. Firstly, the user logs into the primary service and selects a specific subject. The primary service returns a HTML-Frameset as response, where the top-frame contains an HTML-document with the master data of the selected subject. A new instance of a predefined eCRF is generated and the resulting document is displayed using the previously described method. In this process, a chain of HTTP-Requests is generated, in which the user’s credentials are encoded into tokens and distributed to all endpoints to implement SSO.

**Fig. 6:**
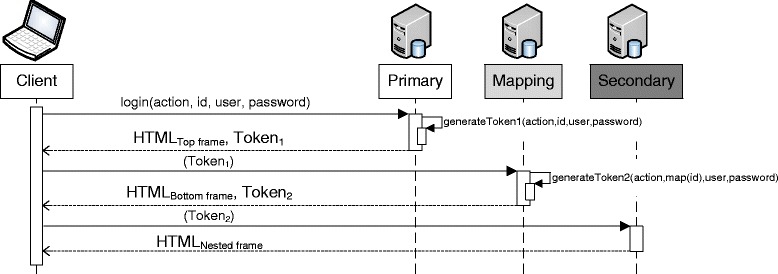
Information flow between services when using HTML Frames. When cascadingly accessing backends, all relevant information is encoded into tokens that are transferred between the hosts via the client. In addition, backends return HTML pages that contain parts of the final frameset. To simplify our illustration, we ignore the fact that the client addresses entities with identifiers from different namespaces than the backends

A basic version of this process is shown in Fig. 6. To simplify our illustration, we assume that the first request, which also logs the user into the system, already contains the ID of the subject for which a new document is to be created. In a real-world scenario, the login process would already have been performed earlier. It can be seen that the user’s credentials and the ID of the data element that is to be displayed are sent to the primary service with the first request. From there on, the operation to be performed, on which data it is to be performed and for which user, is encoded into tokens. These tokens are generated at the backends. This also provides a transparent SSO mechanism. As an alternative to embedding nested frames, this process can also be implemented by using an HTTP-Redirect to route the request from the mapping service to the secondary service.

**Fig. 7:**
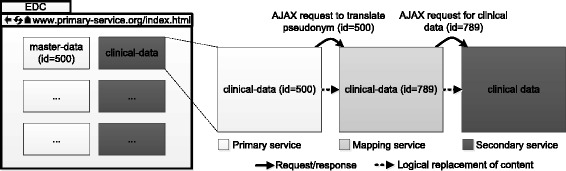
Presentation-layer integration of pseudonymized data with JSONP. First, a HTML document is requested from the primary service, which contains the master data of multiple subjects as well as a set of pseudonyms of related data items for each subject. Via AJAX requests, the pseudonyms are translated to the namespace of the secondary service. Then, the according data is requested, again via AJAX, and the content of the document is updated. To simplify our illustration, we ignore the fact that entities are addressed with identifiers from different namespaces and that the identifiers of entities are encoded into tokens

The second variant of our Web-Mashup uses JSONP requests to display distributed pseudonymized data. It is especially suitable for scenarios in which a larger set of distributed but related entities, e.g., a list of all subjects and an overview of associated clinical data, is to be displayed. The method is sketched in Fig. 7.

First, a HTML-document is delivered to the client by the primary service, e.g. containing the master data of multiple subjects as well as a set of temporary pseudonyms of related data items for each subject. Via JavaScript code, the client then performs a set of AJAX requests to the mapping service in order to translate the pseudonyms from the primary service’s namespace to the namespace of the secondary service. These requests contain a mapping of the actual pseudonyms of the primary service and the temporary pseudonyms. The mapping service associates the temporary pseudonyms with the pseudonyms of the secondary service and returns the mapping to the client. Next, the client requests the data items identified by those pseudonyms from the secondary service. Finally, the temporary pseudonyms in the HTML-document are replaced with the actual data items from the secondary service and the content of the HTML document is updated dynamically. The basic information flow is very similar to the one which is depicted for the method implemented with HTML Frames in Fig. 6. The only difference is that the primary service and mapping service only return tokens, which are then passed to the next receiver, thus implementing the previously described communication channel that is routed via the client.

## Implementations

We have used the described generic solution as a basis for implementing the data management software for several research projects [27–29]. We will focus on two of them [27, 29] which are research networks for rare diseases. Here, the primary actors are health care professionals in an observational study. No specific intervention takes place, and data used for research are collected during health care activities. The associated biobanks use prepared “kits” (tubes with identifiers sent to sites and returned to a central biobank) with pseudonymous labels, which are registered in the system. Internal second level pseudonyms are provided as required by [19, 20]. We will not address the management of biosamples here.

Our first system instance is “mitoRegister”, a multi-site registry which is a part of the mitoNET project [29]. This research network for mitochondrial disorders was started in 2009 under funding by the German Federal Ministry of Education and Research (BMBF). It serves as a platform for over 18 centers in Germany and by August 2015 about 1165 patients have been recruited. Data is managed by 35 eCRFs, which comprise over 900 attributes.

Our second system instance also supports a research network for neurodegenerative diseases, TIRCON [27]. This project was started in 2012, funded by European Commission FP7-Health Work Programme [58]. Our software supports TIRCON’s registry for 13 partners from 8 countries (including the US, UK and Germany). By August 2015 about 265 patients have been recruited. Data is collected in 34 eCRFs consisting of almost 1000 attributes. TIRCON comprises further system parts [27].

In both projects three separated and two-tier pseudonymized data pools are managed by our solution: a) master data, b) clinical phenotype data and c) biospecimen registration data.

Our solution was implemented with Java-Server-Faces as the driving technology for the backends, jQuery for client-side functionalities, MySQL as a database system as well as Tomcat application servers and Apache web servers as runtime environments. Both systems use two-factor authentication with One-Time-Passwords (OTP) following the OATH standard [59] for user accounts with high privileges. Users are provided with time-based dongles that generate short-living passwords, each of which can only be used to access the system exactly once. Communication between endpoints and the clients is secured with Transport Layer Security (TLS/SSL). Automated penetration-tests have been performed and did not detect any weaknesses. Master data is stored encrypted in the according backend. Accountability and integrity are ensured by an audit trail that keeps protocol of every data modification on each backend. We use virtual servers to provide fail-over mechanisms. All endpoints are secured by firewalls. Encrypted backups are created daily and transferred to one dedicated location per backend. Both systems were designed and implemented at our institution in close collaboration with the involved physicians and researchers using an agile development process with short feedback cycles.

Both systems provide web-based data entry, support of cross-validation and plausibility checks, a (logical) central database, an elaborated security concept with multi-tier pseudonymity for patient-, specimen- and image-identifiers. A web-browser is the only software needed to access the system. The informed consent serves as basic agreement for the patient’s research participation. The systems use controlled vocabularies [60] as well as standardized questionnaires [61–63]. Access roles comprise application administrators, monitors, physicians and lab personnel. Each role has different permissions in terms of create-, read-, update- and delete operations (CRUD) for certain types of documents and system objects. Application administrators are able to perform all CRUD-operations on user accounts but do not have access to any type of research data. Monitors may perform read-only operations on clinical data to perform quality assurance. Physicians may perform all CRUD-operations on master data and clinical data. Each physician and patient is associated to his or her home institution. Physicians are only able to access data from patients related to the same institution.

**Fig. 8:**
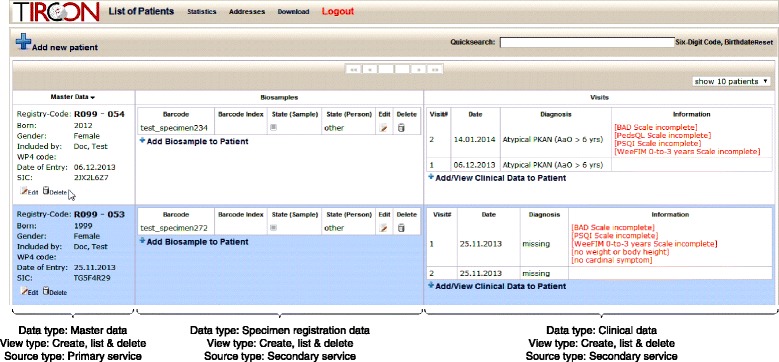
Annotated screenshot of the dashboard of the TIRCON registry. The screenshot shows data about patients included in the registry. The view combines master data with specimen registration data and clinical data. It is realized with JSONP

An example screenshot of the EDC system implemented for the TIRCON project is shown in Fig. 8. Here, a seamless integration of data from different pools is implemented, providing the “*create, list & delete”* functionality defined previously. The view shows an overview and summary data about all subjects, which can be managed by the current user. For each subject, the list is substructured into master data used for re-identification and an overview of the documents used to track biosamples and to collect clinical data. The view is realized with JSONP.

**Fig. 9:**
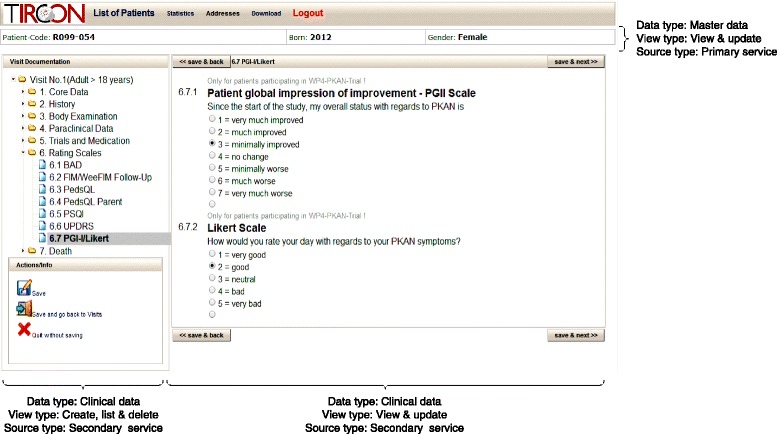
Annotated screenshot of an eCRF implemented in the TIRCON registry. For a selected patient it combines master data with an overview of available documentation and a selected electronic document. The view is realized with HTML-Framesets

A second screenshot from the TIRCON application is presented in Fig. 9. It shows an integrated view of master data and clinical data from an eCRF realized with a HTML-Frameset, which is provided by the primary service. The view implements the previously defined functionality of *view & update*. A top-frame displays the master data of a select subject, whereas the bottom-frame shows the associated documents with clinical data, which are stored at the secondary service. The bottom-frame is organized into two interlinked regions. Firstly a document tree provides an overview of the different documents available for the subject. Secondly, the currently selected document from the tree is displayed.

## Discussion

### Principal results

In this article, we have presented an overview of challenges and solutions for implementing software for the management of pseudonymized data with web technologies. We have described a generic solution that can be tailored to different pseudonymization schemes by using a well-defined subset of the presented techniques. Our approach is independent of the actual distribution of data and it is able to manage associations between patients or visits and further external entities. The aim of our implementation is to build integrated applications, in which the actual distribution of data is transparent to users, providing a virtual central database. Our solution features single-sign-on, supports multi-tier pseudonymity and does not require direct server-to-server communication. By providing various features, our generic solution can be used for the collection of a broad spectrum of different types of data in compliance with national and international laws. Moreover, as a basis, we chose a set of techniques that are supported by modern state-of-the-art browsers as well as legacy browsers. We have shown the practical applicability of our approach, by using it as a basis for implementing two geographically large research networks. Both systems have been in productive use for several years. Several national and international Institutional Review Boards (IRBs) and Data Protection Commissioners of the participating sites have approved the concept.

### Limitations

Current access statistics (i.e. from August 2015) for our applications show that about 25 % percent of our users still access the systems with legacy browsers, such as Internet Explorer 8. As a consequence, we decided to implement our approach with technologies that are supported in older versions of widespread web browsers and did not utilize modern HTML 5 features, such as CORS, or client-side frameworks for building Single-Page Applications, such as AngularJS. Compared to the technologies currently utilized in our implementations, these methods have a great potential to reduce system complexity. The main reason is that instead of distributing business logic over several backend servers, more functionality can be bundled into the client application, reducing the need for logic that orchestrates distributed operations. Moreover, application development and system maintenance are simplified, because the complexity of the backend services can be reduced to a minimum. As support for modern HTML features increases, we plan to upgrade our solution from a tightly coupled application with server-side rendering to a single-page application.

From a security and privacy perspective, current pseudonymization concepts are limited by not being based on risk and threat analyses. This may be the reason why multiple schemes have been proposed but international consensus is missing. Overviews have been provided by [64, 65]; the schemes described differ in their requirements on application level as well as on data level. Some of these differences can be explained with the fact that the schemes have been developed for different use cases (e.g. for data warehouses [66] as compared to research networks [20]). But still, many of the inherent design decisions seem to be ad-hoc and lack thorough justification, which could have been provided by a risk and threat analysis. Some requirements can be well justified with general principles in IT security, e.g., the need-to-know principle and the principle of least privilege. Other methods specified by pseudonymization concepts, however, have a strong impact on system design but lack such justification. Among the important open questions are motivations for the application-level requirements *R-A2* (client-side re-combination only) and *R-A3* (confidentiality of internal identifiers) as well as the data-layer requirement *R-D3* (two-tier pseudonymization).

The general problem is that it remains unclear, how exactly data is to be separated into subsets. The ad-hoc classification into “identifying data” and “other types of data” is insufficient. For example, it is well understood that data which may fall into the second category can be used to re-identify individuals (see [67] for a discussion regarding diagnosis codes). To the best of our knowledge, ISO 25237 [19] is the only work in the context of pseudonymization that lists a set of common identifiers with a high risk of re-identification. But still, no countermeasures against this inherent problem of pseudonymity have been proposed. This situation makes it difficult to find an adequate balance between privacy concerns and support for workflows that require re-identification of data and subjects. For example, the pseudonymization and de-pseudonymization process may be designed differently. The work by Aamot et al. [64] suggests an efficient routine process that requires to contact multiple ombudsmen, each of which controls a horizontal subset (i.e. data about a certain set of patients) of the data, to de-pseudonymize datasets. In contrast, the concept of Pommerening et al. [20] involves two additional parties in the process of de-pseudonymizing research data, each of which controls a vertical subset of the data (i.e. a certain set of attributes for all patients).

### Threats and countermeasures

As already noted, a thorough risk and threat analysis is needed to determine to which extent pseudonymity and related methods, such as multi-tier pseudonymity or client-side re-combination of data, offer protection against common security threats at which costs. This in turn requires an analysis of potential attack vectors, risks associated with common types of data, methods for quantifying re-identification risks, and a consideration of results from related research areas, such as privacy-preserving data publishing or privacy-preserving data outsourcing. An analysis of this kind would exceed the scope of this article. In this article, we do not focus on the methodical basis of pseudonymity, but on its implementation. Analogously to related work [31, 64], we will therefore simply assume that implementing pseudonymity as currently conceptualized offers protection against information disclosure. In the remainder of this section, we will focus on the specific aspects of our implementation and the deployed systems.

The STRIDE [68] methodology provides an appropriate means to analyze threats and countermeasures systematically. STRIDE is an acronym for the security threat types addressed by the methodology which are (1) *spoofing*, (2) *tampering*, (3) *repudiation*, (4) *information disclosure*, (5) *denial-of-service*, and (6) *elevation-of-privilege*. We will relate these principles to the basic security principles of ISO 27000 [69] and RFC-4949 [70]:

1. “*Authenticity* – property that an entity is what it claims to be” [69]
2. “*Integrity* – property of protecting the accuracy and completeness of assets” [69]
3. *“Accountability* – responsibility of an entity for its actions and decisions” [69]
4. “*Confidentiality* – property that information is not made available or disclosed to unauthorized individuals, entities, or processes” [69]
5. “*Availability* – property of being accessible and usable upon demand by an authorized entity” [69]
6. “*Authorization* – approval that is granted to a system entity to access a system resource” [70]

The relation of security principles, threats and implemented countermeasures can be seen in Table 1. Many of the countermeasures deployed and implemented in our systems are well-known and in widespread use. First, we apply hardware-level protection, including restricted access to hardware, secure server rooms with a UPS, and redundant server hardware. Second, we implement network-level measures, such as communication based on TLS with certificates and IP-based filtering of requests. On the host-level, we perform backups and maintain disaster recovery plans, deploy intrusion detection systems, firewalls, virus scanners, perform penetration testing and server hardening and use virtualization as well as automated server updates. On the application-level, our software uses common methods, such as limits for login attempts, automated logout after a certain time period, two-factor authentication, role-based access control, input sanitization (e.g. against SQL injection) and input validation. Additionally, our software implements various pseudonymization methods, as described previously. On the client-level, we employ account management policies and perform user trainings.

**Table 1:** Common threats and countermeasures implemented by our systems

Security principle	STRIDE threat	Countermeasure (deployed)
Authenticity	Spoofing	(1) Non-delegated authentication, (2) TLS with server certificates, (3) Username/password policies, (4) Two-factor authentication,(5) IP-based filtering of requests, (6) One-time access tokens to avoid replay attacks, (7) Limit for login attempts, (8) Penetration testing, (9) Automatic logout after inactivity
Integrity	Tampering	(1) Server hardening, (2) Penetration testing, (3) Intrusion detection system, (4) TLS with server certificates, (5) Software installation policies, (6) Audit trail, (7) Input validation, (8) Penetration testing
Accountability	Repudiation	(1) Auditing and logging
Confidentiality	Information disclosure	(1) Input validation, (2) TLS with server certificates, (3) Access restrictions to server hardware, (4) User training, (5) Encrypted backups, (6) Intrusion detection system, (7) Two-tier pseudonymization, (8) Client-side recombination of distributed data, (9) Encrypted tokens for communication between backends, (10) Penetration testing, (11) Site-based view, (12) Database encryption
Availability	Denial of service	(1) Input validation, (2) IP-based filtering of requests, (3) Virtualization/sandboxing, (4) Redundant server hardware/raid, (5) Backups/disaster recovery plan, (6) Automatic OS updates, (7) Firewalls and virus scanners, (8) Intrusion detection system, (9) Secure server room including UPS and fire extinguisher
Authorization	Elevation of privilege	(1) Role-based Access Control (roles: physician, study nurse, monitor, researcher, lab personnel), (2) Penetration testing, (3) User account management policies, (4) Distributed authorization

Additionally, there are some more-specific security measures implemented by our system. We have covered many of them in the previous sections: prevention of replay attacks on the token infrastructure (one-time access tokens), distributed non-delegated authentication where each component handles authentication and authorization autonomously (distributed authorization), an audit trail that keeps protocol of every data modification on each backend (audit trail) and the encryption of master data in the according backend (database encryption). Additionally, users from a specific participating site are only allowed to access data of patients recruited at their site (site-based view). This is implemented with the role-based access control mechanism.

### Comparison with related work

The work presented in this article is not the first solution that has been proposed for pseudonymized data management. It is one of the very few contributions, however, asking fundamental questions. We have presented a systematic solution for a typical use case, but we strongly suggest further work. Moreover, we have put emphasis on detailed descriptions of alternatives that are available for implementing the methods described in this paper.

There are many articles that focus on application-level aspects of pseudonymization and do not describe technical details about the information systems that manage these data and implement the described processes [71–76]. Some articles on pseudonymization focus on other use cases than our work, leading to different functional requirements. An important group consists of approaches in which re-identification is only supported as an exceptional procedure [66, 77, 78]. In any case, we consider risk and threat analyses a must for the future. Furthermore, we did not consider work in which access to pseudonymized data is controlled by patients, e.g. via smart cards [79, 80].

Several articles have described systems that implement loose coupling. For an in-depth comparison of loosely coupled and tightly coupled architectures we refer to Section “Architectural Options”, but we feel that the most important drawback is that users may need to manually transfer pseudonyms between component systems. The work by Eggert et al. uses a paper-based core process in which pseudonyms are printed on documents [36]. Physicians use the pseudonym from the paper-based documents for remote entry of clinical data. Moreover, a trusted third party is involved in the re-identification process. Demiroglu et al. have published two articles describing loosely coupled systems that implement one-tier pseudonymity [31, 44]. Both systems manage links to two external systems: Starlims [81], which is used for managing biospecimen and secuTrial [82], which acts as a clinical phenotype database.

The most elaborated approach for loose coupling has been presented by Lablans et al. in [26]. Their work describes a reference implementation of a REST-based interface for the realization of clinical research networks. Its main functionality is to support identity management, i.e., to store master data together with an associated pseudonymized link (e.g. identifier) to an external data pool. In the article, the EDC system secuTrial [82] is used as an example. Analogously to our approach, the authors utilize tokens for the communication between the clients and the RESTful backend but these tokens are not cryptographically protected. In contrast to our solution their system only supports one-tier pseudonymization and makes internal pseudonyms (used for storage) visible to users.

The work by Brinkmann et al. [24] is an implementation of the model by Pommerening et al. The system provides an integrated view on data from two separated pools in a web browser. It employs IFrames for a tight coupling of one-tier pseudonymized master data and DICOM images. Temporary identifiers are utilized to integrate these data without making pseudonyms visible to clients. While these design decisions and implementation methods are similar to our solution, it is much narrower in its scope. The system focusses on associating a collection of images with patient master data, which is a rather simple setup with a data model that is not too complex. The system only uses IFrames for providing an integrated view on distributed data. As we have described in Section 2.4.1, IFrames are well suited for simple data structures, but have technological limitations when dealing with more complex data. We have also verified this with experiments. It can therefore be assumed that the system is not able to efficiently provide comprehensive views on complex structures consisting of multiple different entities that are interlinked with high multiplicities. Moreover, the system provides a smaller set of features than ours. Important examples include not supporting multi-tier pseudonymity and not providing alternatives to direct server-to-server communication for the synchronization of temporary identifiers.

Spitzer et al. [25] extend this work by utilizing JSONP to overcome these limitations. The resulting client-side JavaScript library has been published as DSLib [83]. This library is used by the project *Open Source Registry System for Rare Diseases in the EU* (*OSSE)* for seamlessly integrating two data pools [84, 85]. Moreover, the system uses the identity management component by Lablans et al. [26]. The system provides tight coupling of this component with a newly developed EDC system for clinical data. All of these solutions do not support two-tier pseudonymity. Moreover, they focus on integrating master data with clinical phenotype data, whereas our solution is more generic. In our research networks we integrate various types of complex data in several different views (of course, only if permitted and required).

The articles by Bialke et al. [32, 45] describes a loosely coupled approach where generic software modules perform tasks like pseudonymization and record-linkage for research data. The aim is to reduce implementation efforts by providing components that implement standard functionalities necessary in disease registries. The individual modules are hosted together by a Trusted Third Party which provides the according services to external parties. The proposed architecture supports two-tier pseudonymization between master data and clinical phenotype data. The paper [45] focusses mainly on workflow aspects. Both articles do not address presentation-layer integration and integrated user interfaces. Furthermore, they do not discuss technical options for implementation.

## Conclusions

Pseudonymization models are very heterogeneous, already on a conceptual level. Most importantly it remains unclear how exactly data is to be separated into distributed subsets. What is lacking is a thorough risk and threat analysis for pseudonymization schemes, covering at least the data- and the application level. Different architectural solutions exist for managing a set of pseudonymized data subsets, each of which has different properties in terms of usability, support for functional requirements and software complexity. Additionally, these architectures can be implemented with different technologies. In this article, we have analyzed this broad spectrum of architectural options and implementation techniques and we have presented a solution that is generic because it is independent of the actual distribution of data and supports a large set of features. In the future, we will investigate how using more modern HTML features can help to reduce system complexity and thus simplify application development as well as system maintenance.

## Additional file

Additional file 1:
**List of IRBs and ethics committees that have approved the projects described in the article.** (DOCX 61 kb)

## Abbreviations

AJAX: Asynchronous JavaScript and XML
CCOW: Clinical context object workgroup
CORS: Cross-Origin resource sharing
CRUD: Create read update and delete
eCRF: Electronic case report form
EDC: Electronic data capture
FP7: Seventh framework programme
HIPAA: Health insurance portability and accountability act
HL7: Health level seven
HTTP: Hypertext transfer protocol
IFrame: Inline frame
IRB: Institutional review board
ISO: International organization for standardization
JSON: JavaScript object notation
JSONP: JSON with padding
JWE: JSON web encryption
JWK: JSON web keys
JWS: JSON web signatures
JWT: JSON web tokens
MVW: Model-View-Whatever
JS: JavaScript
OATH: Initiative for open authentication
OSSE: Open source registry system for rare diseases in the EU
OTP: One-Time-Password
RBAC: Role-based access control
RDBMS: Relational database management system
REST: Representational state transfer
SAML: Security assertion markup language
SOP: Same-Origin-Policy
SPA: Single-page application
SSL: Secure sockets layer
SSO: Single-Sign-On
TLS: Transport layer security
TTP: Trusted third party
UPS: Uninterruptible power supply
URL: Uniform resource locator
W3C: World Wide Web Consortium
Web API: Web application programming interface
WS: Web service
XML: Extensible markup language
XACML: eXtensible access control markup language
XHR: XMLHttpRequest
