# Supplementary material for: A generic solution for web-based management of pseudonymized data
Source: BMC Med Inform Decis Mak. 2015 Nov 30;15:100. doi: 10.1186/s12911-015-0222-y (PMC4665916; doi:10.1186/s12911-015-0222-y)
Supplement: Additional file 1: — List of IRBs and ethics committees that have approved the projects described in the article. (DOCX 61 kb) [file 12911_2015_222_MOESM1_ESM.docx]

# List of IRBs and ethics committees that have approved the projects described in the article:

- IRB of the Children's Hospital & Clinical Research Center Oakland, Oakland (CA), United States
- Ethics Committee of the University of Munich, Munich, Germany
- Ethics Committee of the University of Belgrade, Belgrade, Serbia
- Ethics Committee of the State Salzburg, Salzburg, Austria
- Ethics Committee of the Ruprecht-Karls University, Heidelberg, Heidelberg, Germany
- Ethics Committee of the Newcastle Upon Tyne Hospitals, Newcastle upon Tyne, United Kingdom
- Ethics Committee of the Martin-Luther-University Halle-Wittenberg, Halle (Saale), Germany
- Ethics Committee of the Hospital Sant Joan de Déu, Esplugues del Llobregat (Barcelona), Spain
- Ethics Committee of the General University Hospital, Prague, Czech Republic
- Ethics Committee of the Friedrich-Wilhelms-University Bonn, Bonn, Germany
- Ethics Committee of the Eberhard-Karls-University Tübingen, Tübingen, Germany
